# Supplementary material for: Food-burying behavior in red imported fire ants (Hymenoptera: Formicidae)
Source: PeerJ. 2019 Jan 25;7:e6349. doi: 10.7717/peerj.6349 (PMC6348953; doi:10.7717/peerj.6349)
Supplement: Supplemental Information 2 — Each test for each colony group of ants that showed active (indicated by “+”) food-transport and food-burying under starved or fed conditions. [file peerj-07-6349-s002.docx]

**Table S1:** Each test for each colony group of ants that showed active (indicated by “**+**”) food-transport and burying under starved or fed conditions.

| Observation | Colony  group | Starved | | | | | | | | |  | Fed | | | | | | | | |
| --- | --- | --- | --- | --- | --- | --- | --- | --- | --- | --- | --- | --- | --- | --- | --- | --- | --- | --- | --- | --- |
|  |  | Real food | | | |  | False food | | | |  | Real food | | | |  | False food | | | |
|  |  | 1 | 2 | 3 | 4 |  | 1 | 2 | 3 | 4 |  | 1 | 2 | 3 | 4 |  | 1 | 2 | 3 | 4 |
| Food-burying | 1 |  |  |  |  |  |  |  |  |  |  | **+** | **+** |  | **+** |  |  |  |  |  |
|  | 2 |  |  |  |  |  |  |  |  |  |  | **+** | **+** | **+** | **+** |  |  |  |  |  |
|  | 3 |  |  | **+** |  |  |  |  |  |  |  | **+** |  | **+** |  |  |  |  |  |  |
|  | 4 |  |  |  |  |  |  |  |  |  |  |  | **+** |  | **+** |  |  |  |  |  |
|  | 5 |  |  |  |  |  |  |  |  |  |  | **+** | **+** | **+** | **+** |  |  |  |  |  |
|  | 6 |  |  |  |  |  |  |  |  |  |  |  |  |  |  |  |  |  |  |  |
|  | 7 |  |  |  |  |  |  |  |  |  |  |  |  |  |  |  |  |  |  |  |
|  | 8 |  |  |  |  |  |  |  |  |  |  |  |  |  |  |  |  |  |  |  |
| Food-transport | 1 | **+** | **+** | **+** | **+** |  |  |  |  |  |  |  |  |  |  |  |  |  |  |  |
|  | 2 | **+** | **+** | **+** | **+** |  |  |  |  |  |  |  |  |  |  |  |  |  |  |  |
|  | 3 | **+** | **+** |  |  |  |  |  |  |  |  |  |  |  |  |  |  |  |  |  |
|  | 4 | **+** | **+** | **+** | **+** |  |  |  |  |  |  | + |  |  |  |  |  |  |  |  |
|  | 5 | **+** | **+** | **+** | **+** |  |  |  |  |  |  |  |  |  |  |  |  |  |  |  |
|  | 6 | **+** | **+** | **+** | **+** |  |  |  |  |  |  |  |  |  |  |  |  |  |  |  |
|  | 7 | **+** |  | **+** | **+** |  |  |  |  |  |  |  |  |  |  |  |  |  |  |  |
|  | 8 | **+** | **+** | **+** | **+** |  |  |  |  |  |  |  |  |  |  |  |  |  |  |  |
