# Supplementary material for: Food-burying behavior in red imported fire ants (Hymenoptera: Formicidae)
Source: PeerJ. 2019 Jan 25;7:e6349. doi: 10.7717/peerj.6349 (PMC6348953; doi:10.7717/peerj.6349)

**Table S2:** Statistical results of repeated measures ANOVAs with time as within-subjects factor and location as between-subjects factors.

1. Assumptions tests

**
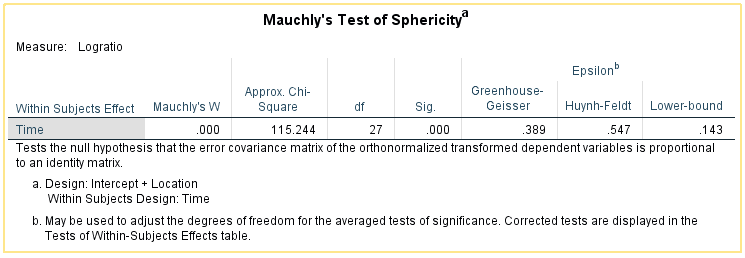
**

Since the Mauchly’s test of sphericity is failed, we will use Greenhouse-Geisser correction for the degrees of freedom.


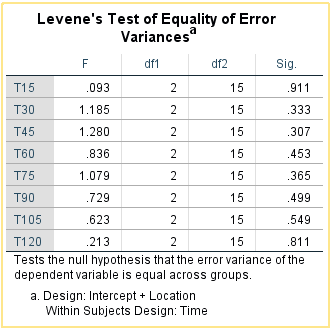


Levene’s test of equality of error variances is passed.

1. Within-subjects effects, between-subjects effects, and interaction

**
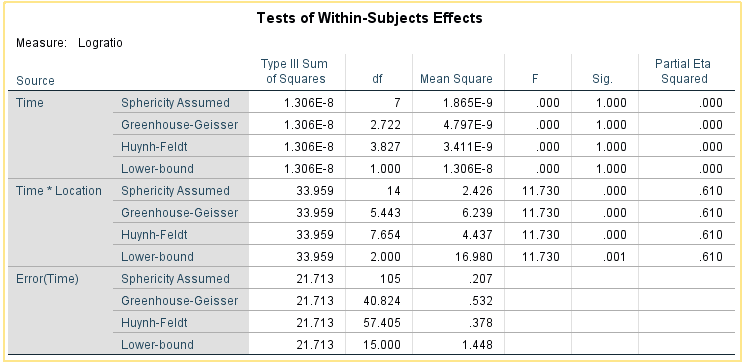
**

Using Greenhouse-Geisser method to adjust the degrees of freedom, the result reveals the significant effect from the interaction between time and location (*F* (5.443, 40.824) =11.730, *P* < 0.001). No significant effect from time is observed (*F* (2.722, 40.824) =.000, *P*=1.000), which makes sense because the average value of percentages of all data in each time interval is equal to 33.33%.


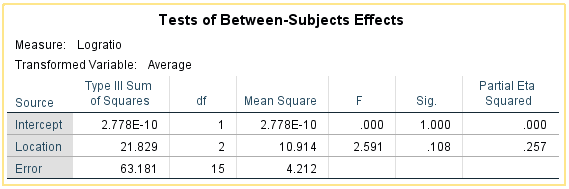


There is no significant effect from location (*F* (2, 15)=2.591, *P*=0.108).

1. **Pairwise Comparison on each Time-interval**

Based on the results in B, we are interested in the comparison of locations on each time-interval. Even though this is a time series, but the effect from time is not significant. We used One-Way ANOVA with Tukey’s Honest Significant Differences (HSD) tests. The corresponding time series figures (Mean ± SE) is presented in Fig. 4b.


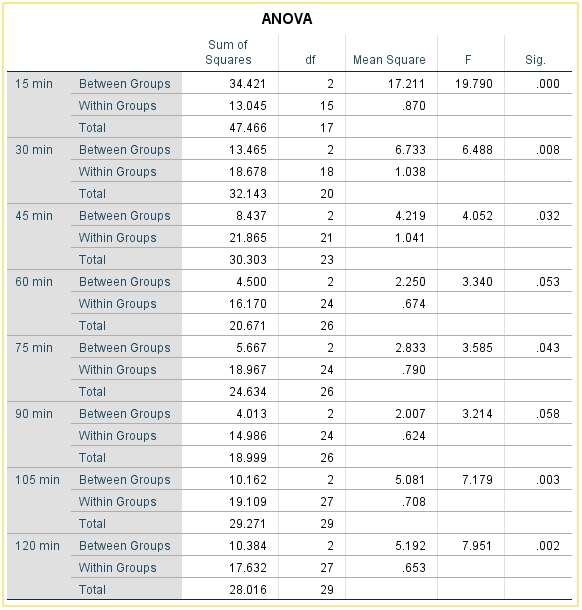


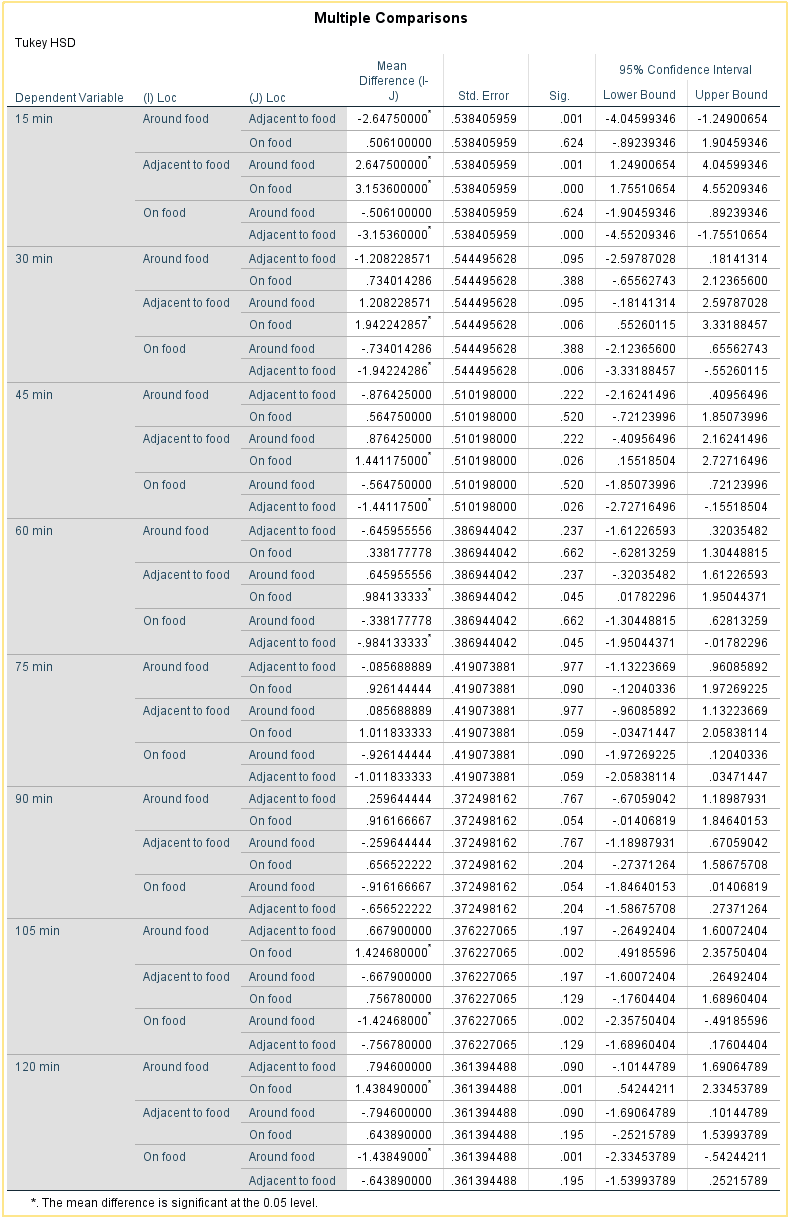

Supplement: Supplemental Information 3 — Statistical results of repeated measures ANOVA with time as a within-subjects factor and location as a between-subjects factor. Using Greenhouse-Geisser method to adjust the degrees of freedom, the result reveals the significant effect from the interaction between time and location (F = 11.730, df = 5.443, 40.824, P < 0.001). No significant effect from time (F = 0.000, df = 2.722, 40.824, P = 1.000) or location (F = 2.591, df = 2, 15, P = 0.108) is observed. We then compared percentages (transformed data) of particles on, adjacent to, or around the food on each time-interval using the one-way ANOVA followed by Tukey’s Honest Significant Differences (HSD) tests. The corresponding time series figures (Mean ± SE) is presented in Fig. 4B. [file peerj-07-6349-s003.docx]
