# Supplementary material for: Food-burying behavior in red imported fire ants (Hymenoptera: Formicidae)
Source: PeerJ. 2019 Jan 25;7:e6349. doi: 10.7717/peerj.6349 (PMC6348953; doi:10.7717/peerj.6349)

**Table S3:** Statistical results of repeated measures ANOVA with time as a within-subjects factor and behavior as a between-subjects factor.

1. Assumptions tests

**
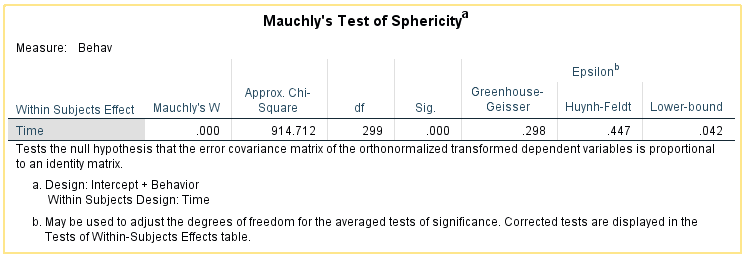
**

Since the Mauchly’s test of sphericity is failed, we will use Greenhouse-Geisser correction for the degrees of freedom.

1. Within-subjects effects, between-subjects effects, and interaction

**
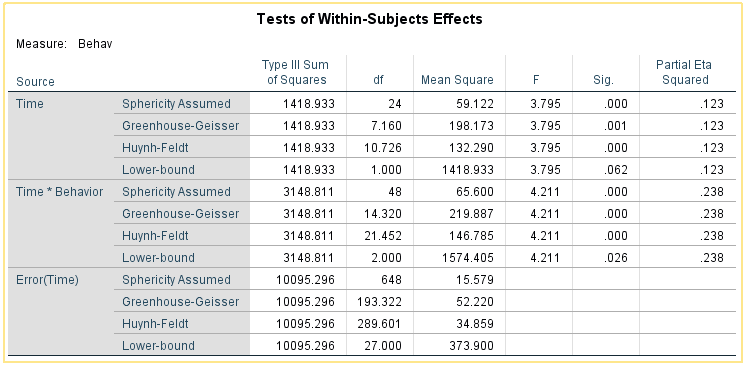
**

Using Greenhouse-Geisser method to adjust the degrees of freedom, the result reveals the significant effect from time (*F* (7.160, 193.322)=3.795, *P*=0.001) and significant effect from the interaction between time and behavior (*F*(14.320, 193.322)=4.211, *P* <0.001).


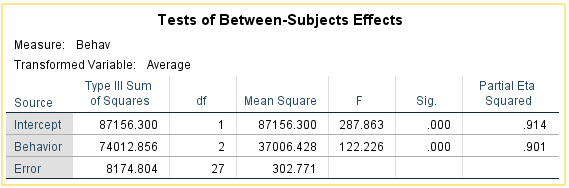


There is a significant effect from behavior (*F*(2,27)=122.226, *P* <0.001).

1. **Pairwise Comparison on each Time-interval**

Based on the results in B, we are interested in the comparison of behaviors on each time-interval. We used One-Way ANOVA with Tukey’s Honest Significant Differences (HSD) tests. The corresponding time series figures (Mean ± SE) is presented in Fig. 5a.


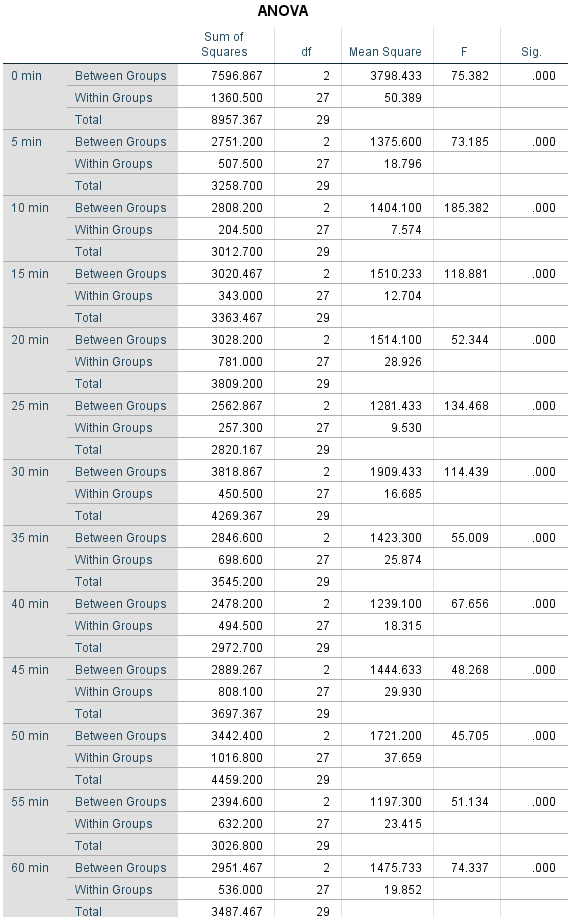


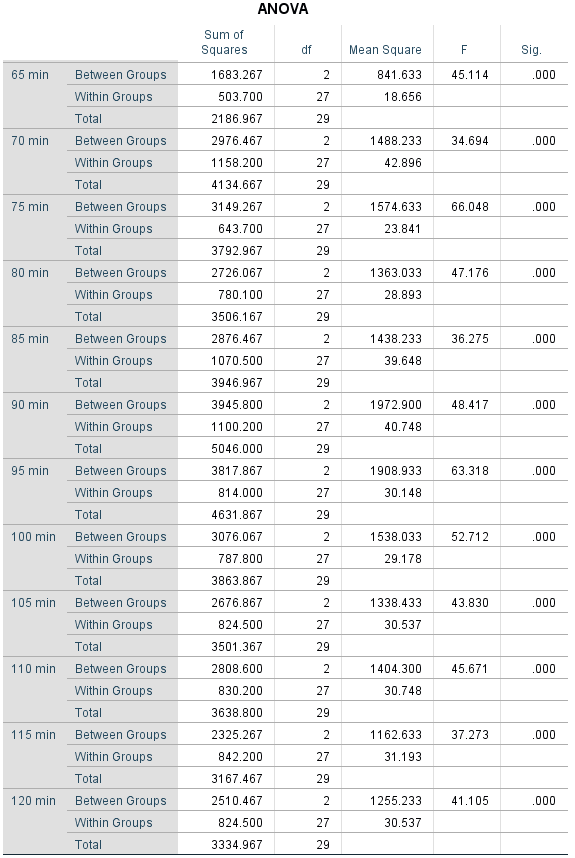


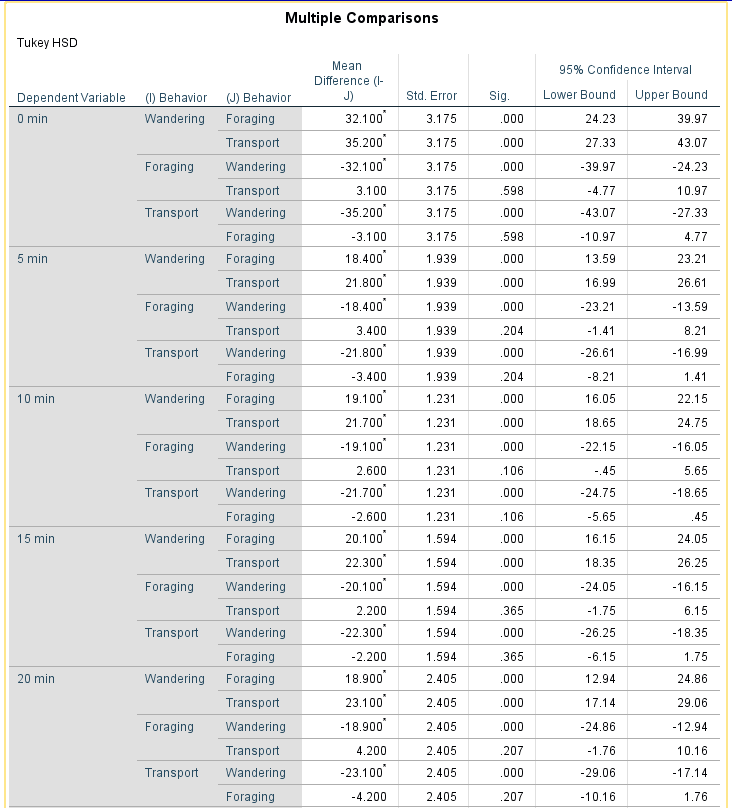


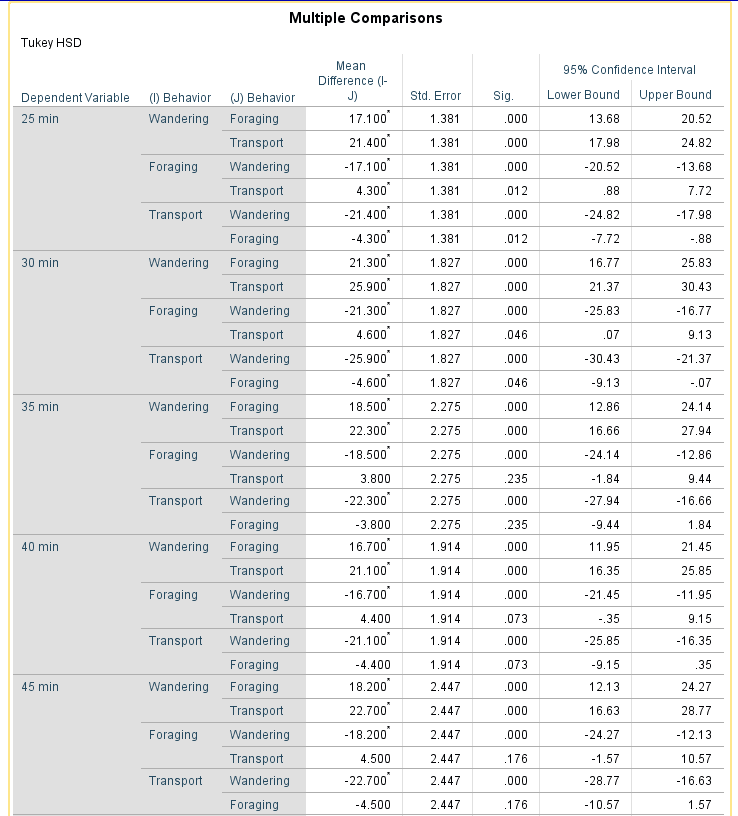


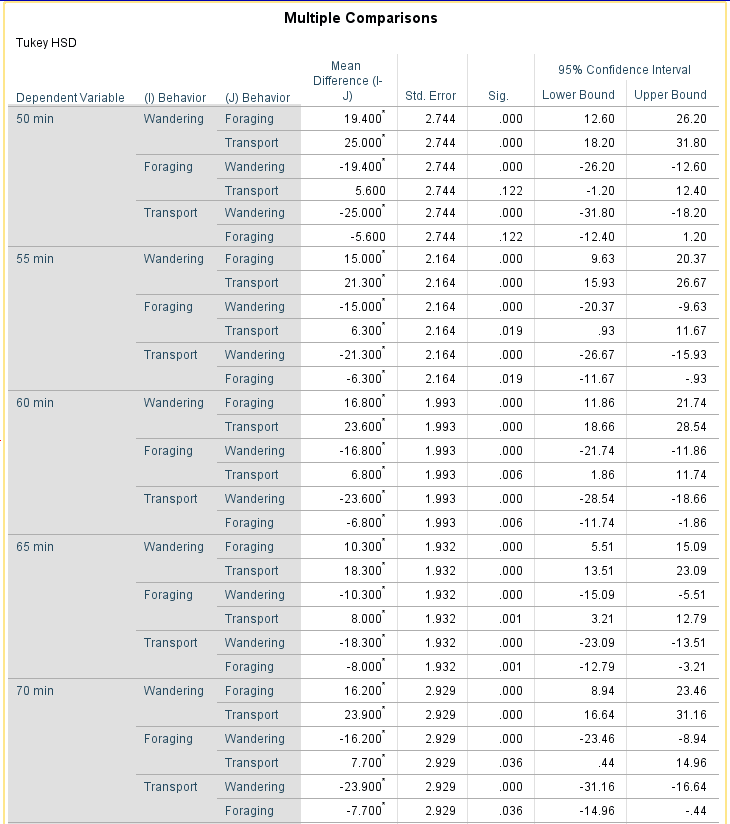


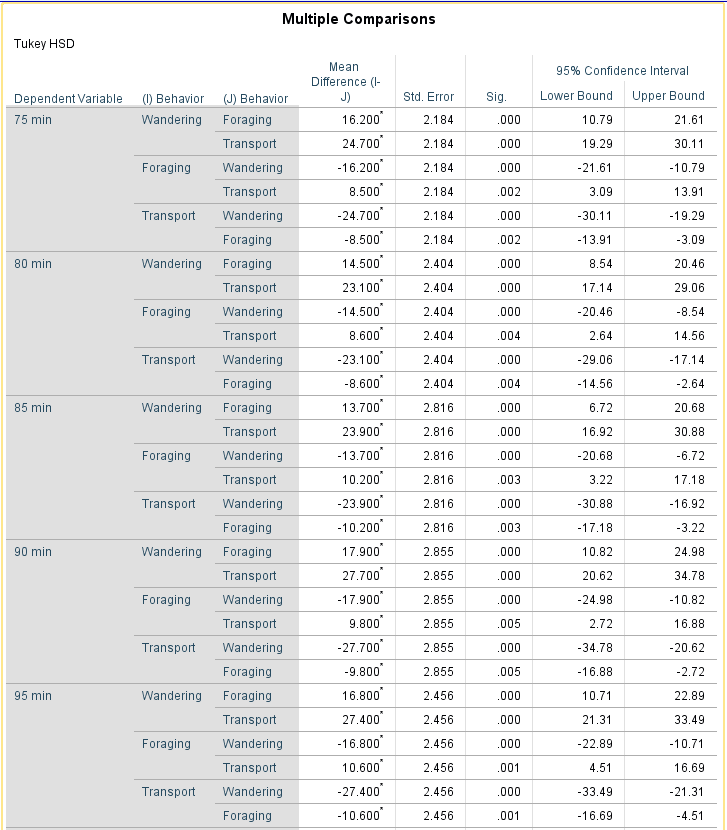


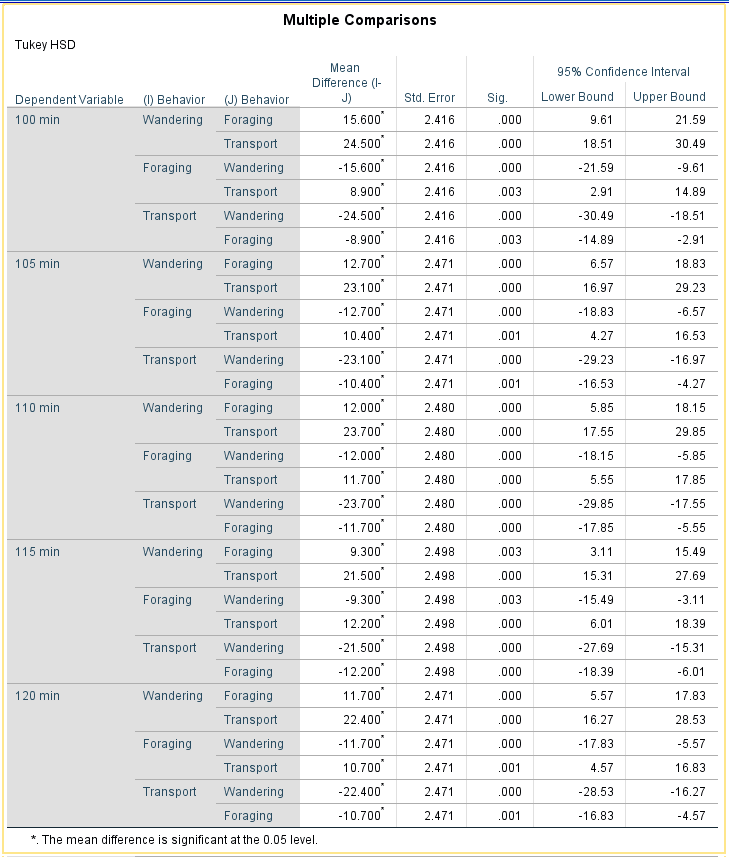

Supplement: Supplemental Information 4 — Statistical results of repeated measures ANOVA with time as a within-subjects factor and behavior as a between-subjects factor. Using Greenhouse-Geisser method to adjust the degrees of freedom, the result reveals the significant effect from time (F = 3.795, df = 7.160, 193.322, P = 0.001), behavior (F = 122.226, df = 2, 27, P < 0.001), and the interaction between time and behavior (F = 4.211, df = 14.320, 193.322, P < 0.001). We then compared the number of Solenopsis invicta workers that exhibited wandering, foraging and food-burying behaviors on each time-interval using the one-way ANOVA followed by Tukey’s Honest Significant Differences (HSD) tests. The corresponding time series figures (Mean ± SE) is presented in Fig. 5A. [file peerj-07-6349-s004.docx]
